# Novel Ti_3_C_2_T_x_ MXene nanozyme with manageable catalytic activity and application to electrochemical biosensor

**DOI:** 10.1186/s12951-022-01317-9

**Published:** 2022-03-09

**Authors:** Rongjun Yu, Jian Xue, Yang Wang, Jingfu Qiu, Xinyi Huang, Anyi Chen, Jianjiang Xue

**Affiliations:** 1 Department of Clinical Laboratory, University-Town Hospital of Chongqing Medical University, Chongqing, 401331 China; 2 School of Public Health and Management, Chongqing Medical University, Chongqing, 400016 China; 3 Department of Clinical Laboratory, First Affiliated Hospital of Guangxi University of Chinese Medicine, Nanning, 530023 China

**Keywords:** Ti_3_C_2_T_x_ MXene, Nanozyme, Electrocatalysis, Cascading catalytic amplification, Electrochemical biosensor

## Abstract

In this work, Ti_3_C_2_T_x_ MXene was identified as efficient nanozyme with area-dependent electrocatalytic activity in oxidation of phenolic compounds, which originated from the strong adsorption effect between the phenolic hydroxyl group and the oxygen atom on the surface of Ti_3_C_2_T_x_ MXene flake. On the basis of the novel electrocatalytic activity, Ti_3_C_2_T_x_ MXene was combined with alkaline phosphatase to construct a novel cascading catalytic amplification strategy using 1-naphthyl phosphate (1-NPP) as substrate, thereby realizing efficient electrochemical signal amplification. Taking advantage of the novel cascading catalytic amplification strategy, an electrochemical biosensor was fabricated for BCR/ABL fusion gene detection, which achieved excellent sensitivity with linear range from 0.2 fM to 20 nM and limit of detection down to 0.05 fM. This biosensor provided a promising tool for ultrasensitive fusion gene detection in early diagnosis of chronic myelogenous leukemia and acute lymphocytic leukemia. Moreover, the manageable catalytic activity of MXene broke a path for developing nanozymes, which possessed enormous application potential in not only electrochemical analysis but also the extensive fields including organic synthesis, pollutant disposal and so on.

**Graphical Abstract:**

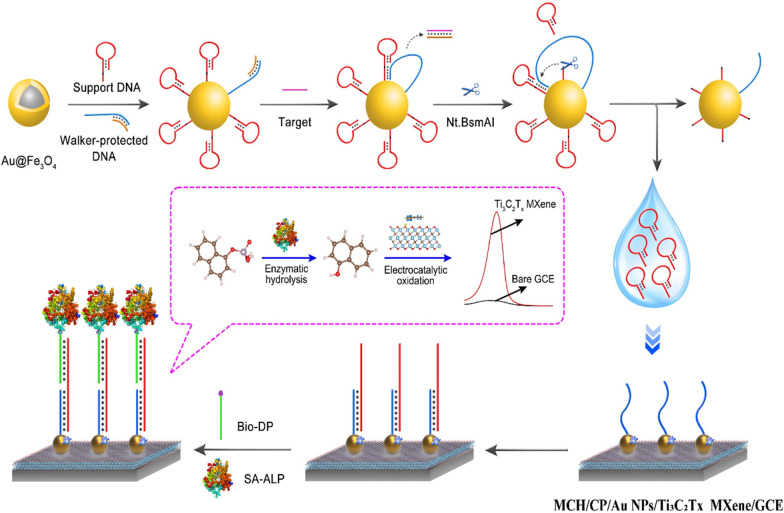

**Supplementary Information:**

The online version contains supplementary material available at 10.1186/s12951-022-01317-9.

## Introduction

Electrochemical biosensor has become one of the most predominant analysis tools in clinical diagnosis due to the outstanding merits of high sensitivity and selectivity, rapid response, low cost, simple instrumentation, easy miniaturization and good quantitative ability [1–4]. Enzymatic electrocatalysis is a widely used technique in electrochemical biosensor, which improves the analytical sensitivity by promoting the electrochemical redox reactions with the help of enzymes [5–7]. Nevertheless, natural enzymes are general cost to manufacture and store, unstable to transfer or modify, and sensitive to harsh physiochemical conditions. Nanozymes, nanomaterials with enzyme-like catalytic activities, well overcome the shortages of natural enzymes [8, 9]. Therefore, nanozymes are attracting increasing attention in bioanalysis to substitute conventional natural enzyme [10, 11]. However, the catalytic activities of conventional nanozymes strongly rely on the defect dependent active centers, such as surface dangling bonds or heterogeneous doping domains, which is distributed unevenly on the surface [12, 13]. As a consequence, the homogeneity of particle is a very important parameter of nanozymes, which brings great challenge to prepare high-quality product and applied to precise quantitative analysis [10]. From this prospective, developing nanomaterials with enzyme-like catalytic activities independent to the morphology or crystal defect is of great significance as this kind of materials might be more easily controlled to obtain uniform catalysis activity.

MXene is termed for a series of two-dimension (2D) transition metal carbides, nitrides, and carbonitrides [14, 15]. Recent years, because typical MXene possess biocompatibility, large specific surface area, rich surface chemistry, tunable lateral size, good electrical conductivity, and mechanical robustness, allowing efficient and selective interaction with target species. MXene-based materials have been utilized as electrocatalysts for detecting small molecules, pharmaceutical drugs, environmental pollutants, and biomarkers. These characteristics render MXene an ideal platform as sensing materials for electrochemical application [16]. The electrocatalytic activity MXene have attracted enormous research interest in diverse fields, such as hydrogen evolution [17], oxygen evolution [18, 19], N_2_-to-NH_3_ conversion [20], fuel cell [21, 22], energy storage [23, 24] and carbon dioxide reduction [25, 26]. These reported works implied a probable reality that the catalysis activities of MXene could be originated from the 2D basal planes rather than the defects, which was significantly different from conventional nanozymes including metallic oxide [27–29], carbon nanomaterials [30, 31], and 2D metallic sulfide [32, 33]. As a result, the electrocatalytic activity of MXene was directly related to the area of flake rather than the shape or morphology. Benefiting from the unique electrocatalytic characteristics and 2D structure, MXenes were expected to provide a chance to easily control the electrocatalytic activity by simply customize the total area of flakes.

**Scheme 1:**
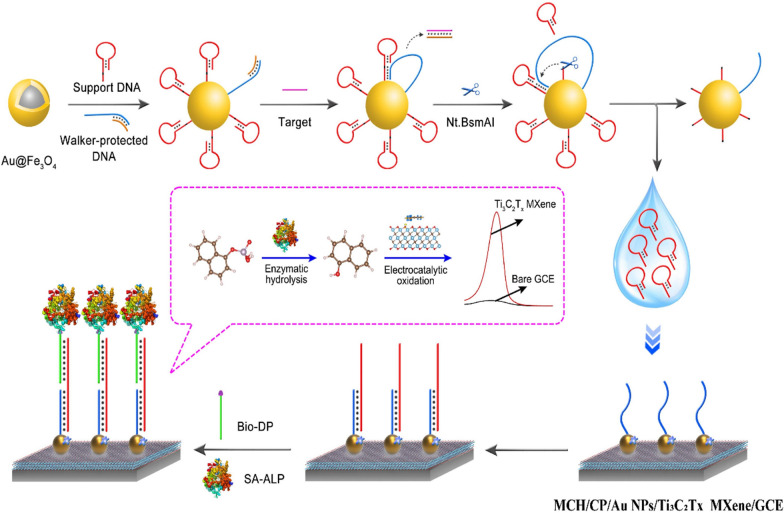
Schematic illustration for the working principle of the electrochemical biosensor

In this work, the electrocatalytic activity of Ti_3_C_2_T_x_ MXene for phenols oxidation was identified and applied to constructing a cascading catalytic amplification strategy for electrochemical biosensor to determine of BCR/ABL fusion gene, the key biomarker for clinical diagnosis of chronic myeloid leukemia (CML). Ti_3_C_2_T_x_ MXene presented efficient and area-dependent phenol adsorption on the 2D plane, thus catalyzing the electrochemical oxidation. For biosensor application, Ti_3_C_2_T_x_ MXene was spread on electrode and further decorated with gold nanoparticles for DNA capture probe (CP) immobilization. Besides, DNA walking machine was employed to recognize target BCR/ABL fusion gene and mediate nucleic acid amplification. As illustrated in Scheme 1, the DNA walking machine would start DNA nicking and expose DNA fragments from the magnetic beads in the presence of BCR/ABL fusion gene. The exposed DNA fragments helped the assembly of biotin labeled DNA probe (Bio-DP) on the sensing surface according to the sandwich DNA hybridization of CP-DNA fragment-Bio-DP. Finally, streptavidin modified alkaline phosphatase (SA-ALP) further was modified onto the biosensing interface via the specific biotin-streptavidin reaction. With the addition of 1-naphthyl phosphate (1-NPP) in the electrolyte solution, 1-naphthol was produced via ALP-catalytic hydrolysis of 1-NPP and generated an amplified electrochemical signal via Ti_3_C_2_T_x_ MXene-catalytic electrochemical oxidization. With DNA walking machine and cascading catalysis for signal amplification, the electrochemical biosensor achieved excellent sensitivity for detection of BCR/ABL fusion gene with the linear range from 0.2 fM to 20 nM and limit of detection down to 0.05 fM, which could provide a powerful bioanalysis tool for clinical diagnose of CML. Moreover, the efficient electrocatalysis activity of Ti_3_C_2_T_x_ MXene for phenols oxidation possessed great application potential in the more fields including sewage treatment and organic synthesis and so on.

## Experimental section

### Reagents and materials

Ti_3_C_2_T_x_ (MXene) few layer dispersion solution (Lateral size 2–5 µm), Fe_3_O_4_ nanoparticles, TiO_2_ nanoparticles, bulk Ti_3_AlC_2_ and WS_2_ nanosheets (Diameter 2–5 µm) were obtained from Jiangsu XFNANO Materials Tech. Co., Ltd. (Nanjing, China). MoS_2_ nanosheets (Diameter 20–500 nm) were obtained from Nanjing JCNANO Tech. Co., Ltd. (Nanjing, China). NH_2_-Fe_3_O_4_ and Nafion solutions were obtained from Aladdin Biochemical Tech. Co., Ltd. (Shanghai, China). Gold chloride (HAuCl_4_∙4H_2_O), sodium citrate, 6-mercaptohexanol (MCH), 1-naphthyl phosphate (1-NPP), 1-naphthol, streptavidin–alkaline phosphatase (SA-ALP), 4-nitrophenol, β-estradiol and diethanolamine (DEA) were purchased from Sigma-Aldrich Chemical (St. Louis, USA). Tris(2-carboxyethyl) phosphine hydrochloride (TCEP) was purchased from Sangon Biotech. Co., Ltd. (Chongqing, China). Nt.BsmAI nicking endonuclease (Nt.BsmAI) and CutSmart buffer were provided by New England Biotech. Co., Ltd. (Beijing, China). All high-performance liquid chromatography (HPLC)-purified sequences (Additional file 1: Table S1) in our experiments were ordered from Sangon Biotech. Co., Ltd. (Shanghai, China). Clinical serum samples were obtained from the University-Town Hospital of Chongqing Medical University (Chongqing, China). The buffers and solutions involved in this experiment were display in Additional file 1: S1.

### Modification of electrode surface

Prior to modification, the bare glassy carbon electrode (GCE) was polished with 300 nm and 50 nm alumina slurries to a mirror-like surface and then rinsed ultrasonically with ultrapure water, anhydrous ethanol and ultrapure water for 5 min in sequence. Then, the GCE was dried by nitrogen at room temperature. Simultaneously, Ti_3_C_2_T_x_ MXene (0.1 mg/mL) was suspended in ultrapure water containing a 0.1% Nafion solution and sonicated for 60 min. Gold nanoparticles (AuNPs) were synthesized according to a typical method, and the detailed procedure was elaborated in Additional file 1: S3. The products were stored at 4 °C protected from light for further use.

Next, 10 µL of the Ti_3_C_2_T_x_ MXene (0.10 mg/mL) suspension was dropped onto the GCE surface and allowed to dry slowly, followed by the addition of 10 µL AuNPs solution and drying at room temperature to obtain the modified electrodes (AuNPs/Ti_3_C_2_T_x_ MXene/GCE). Afterwards, 10 µL of thiolated capture probe (CP) pretreated by TCEP was dropped onto the AuNPs/Ti_3_C_2_T_x_ MXene/GCE surface and incubated overnight at 4 °C. After being washed with washing buffer, the modified electrode was further incubated with 1.0 mM MCH for 1 h at room temperature to block the nonspecific site, obtaining the electrochemical biosensing platform (MCH/CP/AuNPs/Ti_3_C_2_T_x_ MXene/GCE).

### Preparation of DNA walking machine

DNA walking machine was prepared with reference to a previous report [34]. First, 1.0 µL walker probe solution (2.0 µM) and 1.0 µL protecting probe solution (2.0 µM) were mixed and heated at 95 °C for 5 min, and then naturally cooled to obtain ds-DNAs. Later, 20 µL support probe solution (2.0 µM) was sufficiently mixed with dsDNA and added to 20 µL Au@Fe_3_O_4_ (the detailed procedure was described in Additional file 1: S4), stirred overnight and magnetically separated to obtain the expected DNA walking machine (DNA-Au@Fe_3_O_4_). Finally, the well prepared DNA walking machine was further suspended in PBS and stored at 4 °C for further use.

### Procedure for BCR/ABL fusion gene detection

First, BCR/ABL fusion gene standard sample (1.0 µL) at different concentrations, 10 U Nt.BsmAI and were added in to the dispersion of DNA walking machine and kept for 2 h at 37 °C. Then, supernatant solution was collected numerous after magnetic separation, which contained the produced intermediate DNAs. After that, 10 µL of the above supernatant solution and 10 µL of 2.5 µM biotinylated detection probe were dipped onto the electrode and incubated at 37 °C for 1 h. Rinsing with washing buffer, the obtained electrode was treated in 10 µL of DEA buffer containing 1.25 µg/mL SA-ALP and 8 mg/mL BSA at 37 °C for 30 min. Finally, the electrochemical signal was measured in the DEA buffer containing 1.0 mg/mL 1-NPP by differential pulse voltammetry (DPV) after rinsing with DEA buffer to remove the unbound SA-ALP. All parameter configurations of electrochemical measurements were shown in Additional file 1: S5.

### Theoretic calculation methods

The first principle calculations are performed by Vienna Ab initio Simulation Package (VASP) [35] with the projector augmented wave (PAW) method [36]. The exchange-functional is treated using the Perdew-Burke-Ernzerhof (PBE) [37] functional, in combination with the DFT-D correction [38]. The cut-off energy of the plane-wave basis is set at 500 eV. For the optimization of both geometry and lattice size, the Brillouin zone integration is performed with 2*2*1 Monkhorts-Pack k-point sampling. The self-consistent calculations apply a convergence energy threshold of 10^–5^ eV. The equilibrium geometries and lattice constances are optimized with maximum stress on each atom within 0.02 eV/Å.

## Results and discussion

### Morphological and elemental analysis of the Ti_3_C_2_T_x_ MXene

**Fig. 1:**
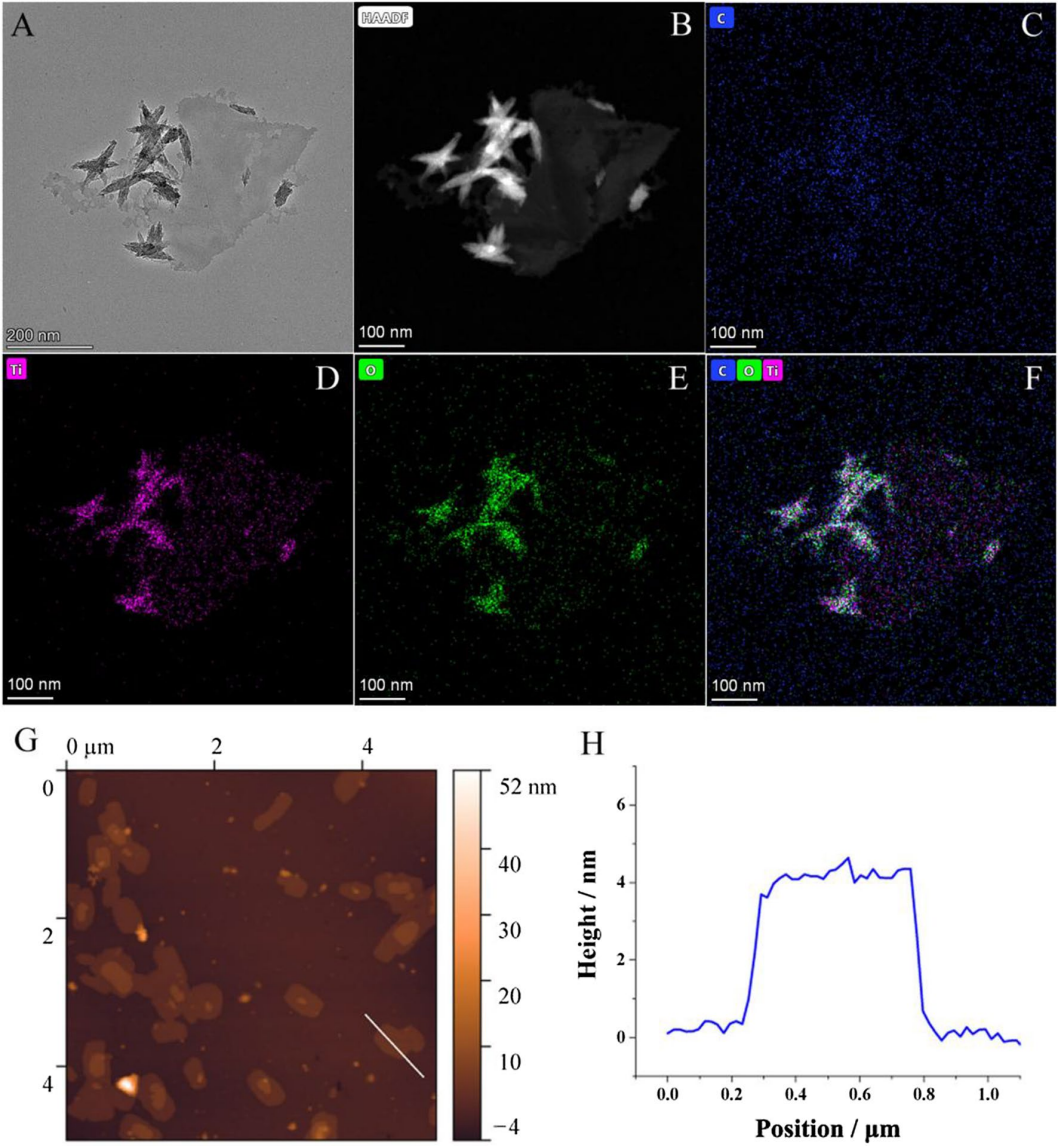
**A** TEM image, **B** HAADF-STEM image and **C**–**F** STEM-EDS elemental mappings of the Ti_3_C_2_T_x_ MXene flakes. **G** AFM image of the Ti_3_C_2_T_x_ MXene flakes and **H** the height profile along the white line

Transmission electron microscopy (TEM) was employed to study the morphology of the used Ti_3_C_2_T_x_ MXene sample, which presented a remarkably large flake and some stacked little fragments (Fig. 1A). Moreover, the high-angle annular dark-field (HAADF)-STEM image showed no observable spot on the flake, suggesting the uniform distribution of the elements (Fig. 1B). As shown in Fig. 1C–E, STEM-EDS elemental mappings of C, Ti, and O presented outlines well matched with the HAADF-STEM image, which visually displayed the elemental composition of the Ti_3_C_2_T_x_ MXene.

Energy dispersive X-ray spectroscopy (EDX) was also utilized to analyze the elemental composition of the Ti_3_C_2_T_x_ MXene (see in Additional file 1: Fig. S1), which involved of C, O, Ti, F and Al elements. Among them, F and Al were mainly from the residual impurities and their contents were notably lower than C, O, and Ti.

Atomic force microscope (AFM) was employed to further study the morphology of Ti_3_C_2_T_x_ MXene. As shown in Fig. 1G and 1H, AFM image presented sheets with thickness of about 4 nm, corresponding to the thickness of 3 layers.

### Electrocatalytic activity of Ti_3_C_2_T_x_ MXene for phenolic compound oxidation

**Fig. 2:**
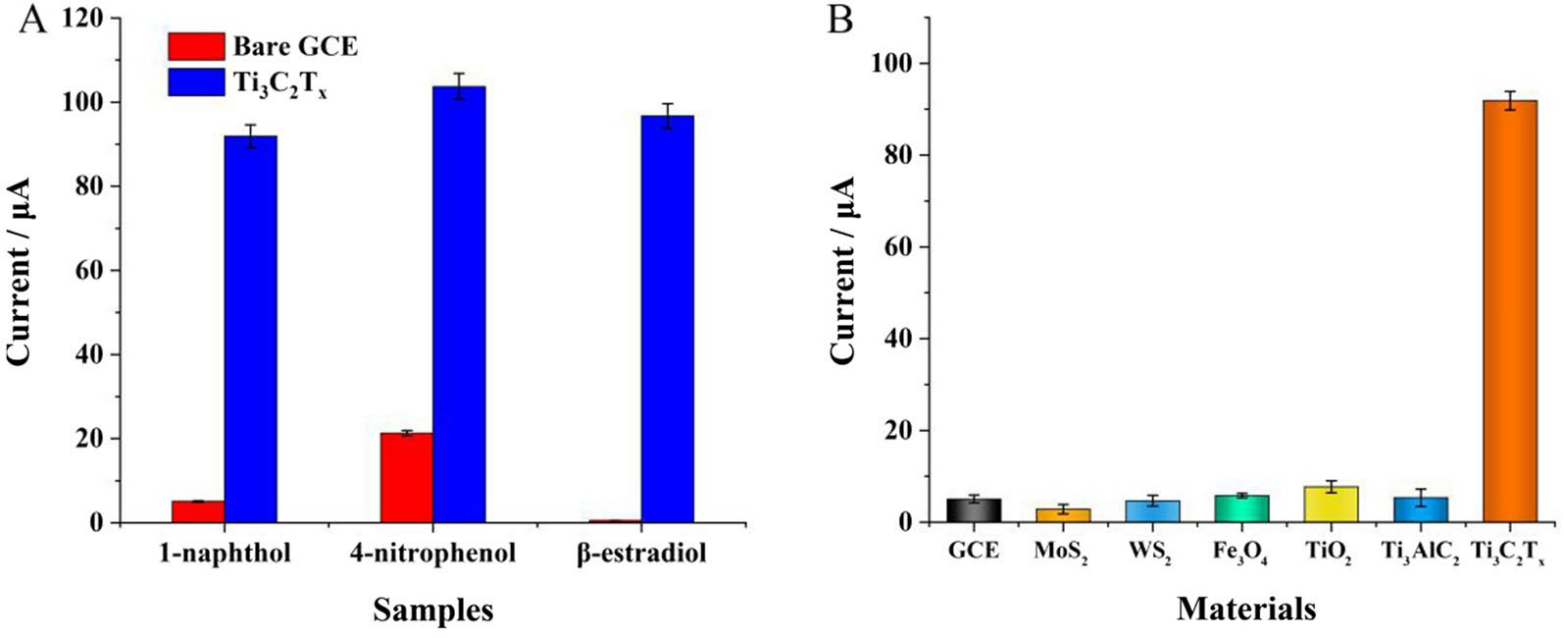
**A** Oxidation currents of different phenolic samples measured with bare GCE and Ti_3_C_2_T_x_ MXene modified electrode, respectively; **B** Oxidation current of 1-naphthol measured with different materials modified electrodes

The electrocatalytic activity for phenolic compound oxidation of Ti_3_C_2_T_x_ MXene was confirmed by testing the electrocatalytic performances with different phenolic substrates, including 1-naphthol, 4-nitrophenol, and β-estradiol. As shown in Fig. 2 the modification of MXene significantly improved the oxidation currents for all the three phenolic compounds (DPV curves were seen in Additional file 1: Fig. S2), indicating the favourable and comprehensive electrocatalytic activity of Ti_3_C_2_T_x_ MXene for phenolic compound oxidation. Moreover, it's notable that the oxidation peaks presented distinct shifts to lower potential, revealing the electron transfer between Ti_3_C_2_T_x_ MXene and phenolic compound.

To further profile the unique electrocatalytic activity of Ti_3_C_2_T_x_ MXene, several nanomaterials including MoS_2_ nanosheets, WS_2_ nanosheets, Fe_3_O_4_ nanoparticles, TiO_2_ nanoparticles and bulk Ti_3_AlC_2_ were separately employed as constrast with 1-naphthol as substrate. It's notable that only Ti_3_C_2_T_x_ MXene presented observable electrocatalytic activity for 1-naphthol oxidation, indicating that the unique electrocatalytic activity was the intrinsic property of Ti_3_C_2_T_x_ MXene.

**Fig. 3:**
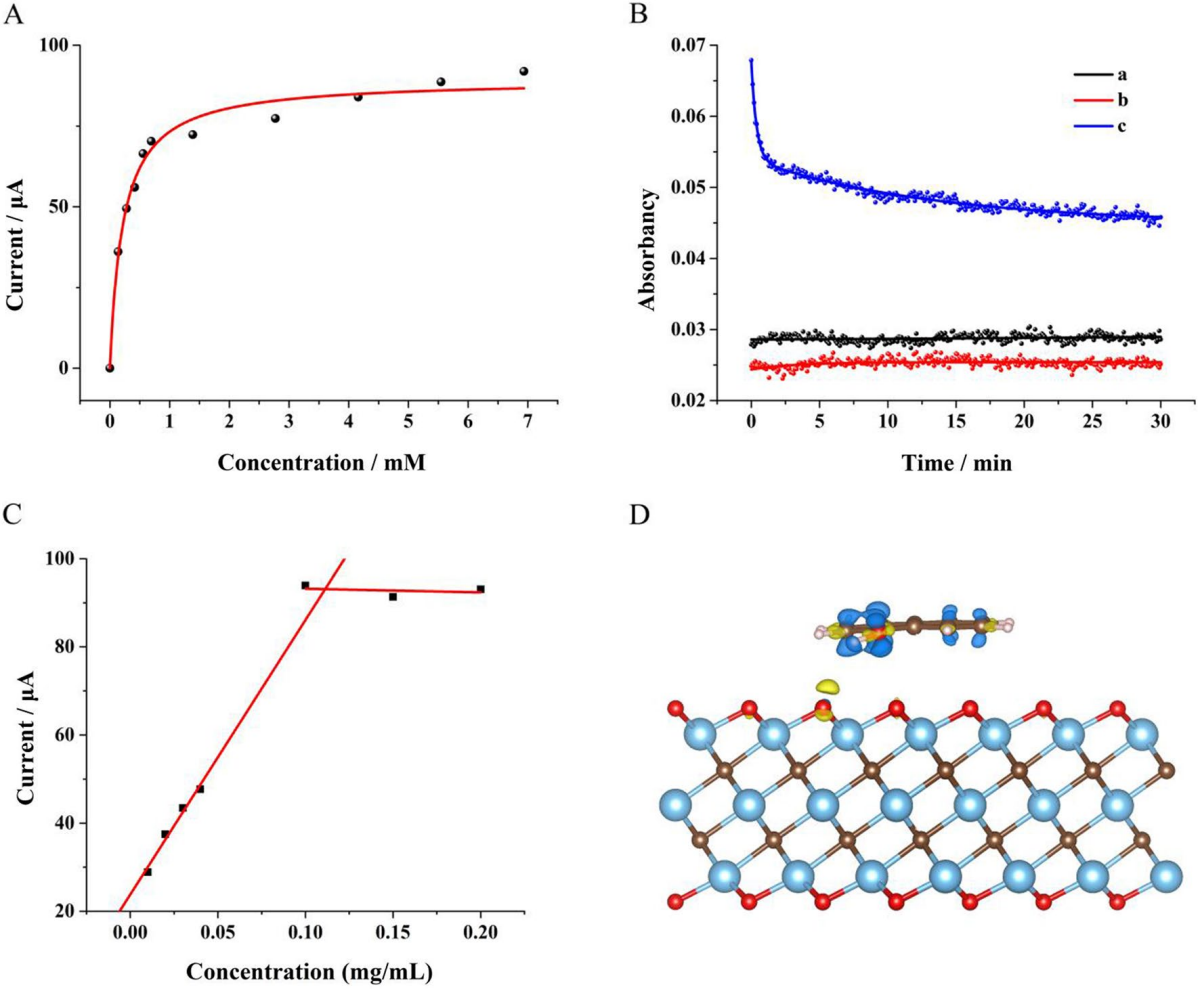
**A** Oxidation currents of 1-naphthol at different concentrations measured with Ti_3_C_2_T_x_ MXene modified electrode; **B** Absorbancy at 387.5 nm of 1-naphthol solution (curve a), mixture of 1-naphthol and hydrogen peroxide (curve b), mixture of 1-naphthol, hydrogen peroxide and Ti_3_C_2_T_x_ MXene (curve c), respectively. **C** The Faradic currents measured by electrode modified with Ti_3_C_2_T_x_ MXene at different concentrations in solution containing 7.0 mM 1-naphthol. **D** Charge density difference of the "lying-down" adsorption mode. Yellow and blue represent charge accumulation and depletion (isovalue: 0.005 au)

To quantitatively present the electrocatalytic activity of Ti_3_C_2_T_x_ MXene for 1-naphthol oxidation, DPV curves were measured with the addition of 1-naphthol at different concentrations. As shown in Fig. 3A, the peak current increased with the increasing concentration of 1-naphthol at low concentrations until reaching about 90 μA. According to Faraday's laws of electrolysis and Michaelis–Menten equation, the fitting curve was achieved with hyperbola function, where the Michaelis constant was calculated to be 0.22 mM, indicating that Ti_3_C_2_T_x_ MXene possessed strong affinity to 1-naphthol [39]. Moreover, the catalytic activity of Ti_3_C_2_T_x_ MXene for 1-naphthol oxidation in homogeneous phase solution was explored using hydrogen peroxide as oxidant. Figure 3B displayed the real-time absorbance of the aqueous solutions at 387.5 nm, including 1-naphthol (curve a), mixture of 1-naphthol and hydrogen peroxide (curve b), mixture of 1-naphthol, hydrogen peroxide and Ti_3_C_2_T_x_ MXene (curve c), respectively. It could be seen that the reaction ratio was significantly improved with the addition of Ti_3_C_2_T_x_ MXene, indicating that Ti_3_C_2_T_x_ MXene could efficiently catalyze the 1-naphthol oxidation by hydrogen peroxide as well [40].

To survey the quantitative relation between electrocatalytic activity and amount of Ti_3_C_2_T_x_ MXene, Ti_3_C_2_T_x_ MXene solutions at different concentrations were employed to modify GCE to test the electrocatalytic activities for 1-naphthol oxidation, respectively. As shown in Fig. 3C, the Faradic current increased linearly with the increasing concentration of Ti_3_C_2_T_x_ MXene in low concentration range and reached a constant in high concentration range, indicating that the electrocatalytic activity was in direct proportion to the total modified area of Ti_3_C_2_T_x_ MXene flakes but not further improved by the stacking of the flakes.

### Mechanism study for the catalytic activity of Ti_3_C_2_T_x_ MXene

The first principle calculations are performed to explain the catalytic activity of Ti_3_C_2_T_x_ MXene for 1-naphthol oxidation. The free energies of 1-naphthol adsorption were first calculated to determine whether 1-naphthol could be adsorbed by Ti_3_C_2_T_x_ MXene. The optimized geometries of 1-naphthol on and Ti_3_C_2_T_x_ MXene were given in Fig. S3 (see in Additional file 1), which showed 1-naphthol molecule adsorbed on the plane surface of Ti_3_C_2_T_x_ MXene in a "lying-down" or "standing-up" manner, with adsorption energies of − 1.18476 and − 0.75625 eV, respectively, indicating relative strong physical adsorption. As a result, the "lying-down" adsorption of 1-naphthol on Ti_3_C_2_T_x_ MXene was critical in the electrocatalytic oxidation. To further investigate the origin of 1-naphthol adsorption, the differential charge density of 1-naphthol adsorbed on Ti_3_C_2_T_x_ MXene was calculated. As shown in Fig. 3D, the changes in charge density caused by 1-naphthol adsorption mainly came from hydroxyl group and oxygen atoms. Moreover, changes in charge density were also found on the aromatic rings, indicating that the aromatic structure played a role in leading to the "lying-down" adsorption mode of 1-naphthol on Ti_3_C_2_T_x_ MXene.

### Characterization of the DNA walking machine

To quantify the efficiency of DNA Walking machine, fluorescence dynamics experiments were carried out to verify it. Firstly, AuNPs and ssDNA-functionalized AuNPs were prepared according to previous literature with little adjustment [41]. In short, sodium citrate (3 mL, 1%) was added rapidly to the boiling solution of HAuCl_4_ (100 mL, 1%). After the color changed from pale yellow to wine-red, the mixture was stopped heating and cooled to room temperature (RT) with continued stirring. The AuNPs had an average particle size of 13 nm and were stored at 4 °C for further use. The preparation method of ssDNA (support probe and walker-protect dsDNA) -functionalized AuNPs is as follows. Firstly, the 1428 μL denature-supporting probe (2 μM) and 72 μL denature-walker-protect dsDNA (2 μM) were mixed with 1 μL acetic acid (500 mM, pH 5.2) and 0.5 μL TCEP (100 mM) at RT for 1 h, respectively. Then the mixture was added into 1 mL AuNP solution, and the resultant solution was stored in a drawer at RT for at least 16 h. After 25 μL Tris–acetate (500 mM, pH 8.2) was added to the mixture, 250 μL NaCl (1 M) was dropwise added to the mixture every three hours (30, 40, 50, 60, 70 μL were added respectively). Subsequently, the resulting mixture was stored in a drawer overnight. Lastly, the mixture was centrifuged (10 000 rpm, 10 min) to remove the excess reagents, and the red precipitate was washed and dispersed in DNA preparation solution for further use.

**Fig. 4:**
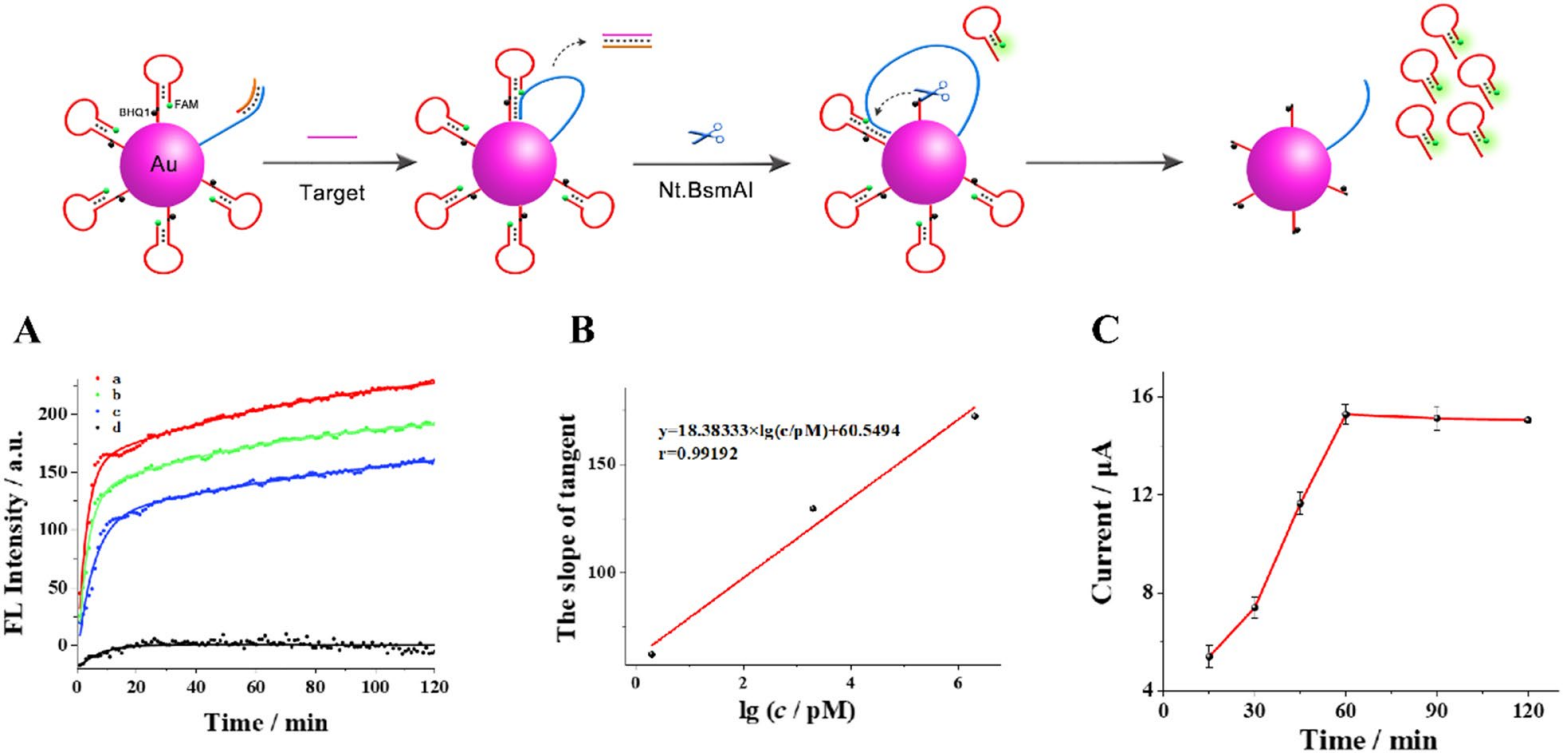
**A** Fluorescence kinetics curves of the nanomachine with addition of 10 U Nt.BsmAI and target BCR/ABL fusion gene at different concentrations (from a to d: 2 μM, 2 nM, 2 pM and blank, separately). **B** The relationship between the slope of tangent at t = 0 s and logarithmic value of target BCR/ABL fusion gene concentrations (range of concentration: 2 pM, 2 nM, and 2 uM separately). **C** Time optimization of intermediate DNAs hybridizing with the capture DNA

Secondly, fluorescence kinetics curve was shown in Fig. 4A to prove the cutting efficiency of Nt.BsmAI nicking endonuclease (Nt.BsmAI). The curve a, b and c showed corresponding changes when different concentrations of target BCR/ABL fusion gene and 10 U Nt.BsmAI were added into the ssDNA (support probe and walker-protect dsDNA) -functionalized AuNPs solution, respectively. Curve d was the blank control. The slope of the curve reflects the reaction rate of enzyme shearing, which is correlated with the concentration of target gene. As can be seen from the figure, the reaction rate is fast. At the same time, when the reaction reached 2 h, the shearing enzyme still did not reach the maximum shearing value, which means that the shearing enzyme has not been completely reacted. Thus, the cutting efficiency of the Nt.BsmAI is excellent. In addition, the time of releasing hairpin structure DNA can also be known from the curve. The sharply rising stage in the curve mainly the enzymatic cleaving on the prehybridized Support DNA-Walker. It can be seen from the figure that it takes about 6–8 min to cleave and release the hairpin. The slope of tangent at t = 0 s was calculated with the fitting curves indicating the cleaving rate was highly related to the concentration of substrate in Fig. 4B. The slope of tangent at t = 0 s and logarithmic value of target BCR/ABL fusion gene concentrations presents well linear dependence range from 2 pM to 2 μΜ with pearson correlation coefficient of 0.99192, which is corresponding to the kinetic characteristic of first-order reaction.

Thirdly, as for the time of hairpin structure intermediate DNAs hybridized with the thiolated capture DNA on the electrode surface to form the sensing interface, we also conducted relevant optimization experiments. As shown in Fig. 4C, the time needed was only 1 h.

Lastly, we have consulted relevant literature, which shows that the amount of DNA loading on 15 nm gold nanoparticles is 20–30 when the concentration of NaCl is 140 mM [42]. According to the concentration and dosage of AuNPs, the amount of ssDNA fabricated on the interface of this sensor was calculated to be about 1 × 10^10^.

### Optimization of experimental conditions

**Fig. 5:**
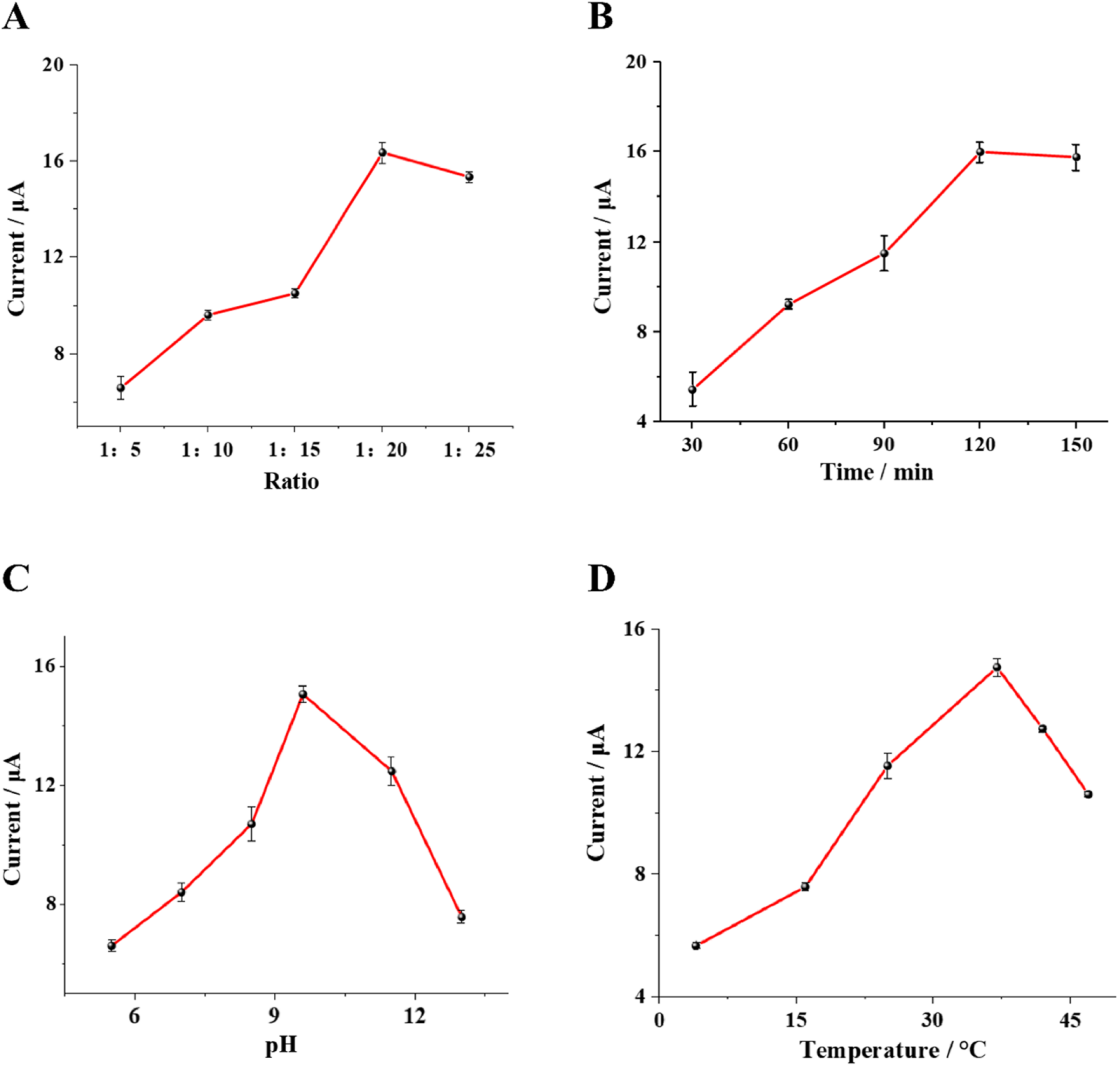
Optimization of experimental conditions: **A** ratio of walker to support DNA, **B** cleaving time of Nt.BsmAI nicking endonuclease, **C** pH of DEA buffer, **D** cleaving temperature of Nt.BsmAI nicking endonuclease. The error bars represent the standard deviation of three parallel measurements

To achieved optimal analytical performance of the biosensor, some critical experimental conditions were optimized, including the ratio of walker probe to support probe, the cleaving time of Nt.BsmAI nicking endonuclease, the pH of DEA buffer and the cleaving temperature of Nt.BsmAI nicking endonuclease. As shown in Fig. 5, optimal ratio of walker probe to support probe, cleaving time, pH of DEA buffer and temperature were achieved to be 1:20, 120 min, 9.6 and 37 °C, respectively.

### Analytical performance of the proposed electrochemical biosensor

**Fig. 6:**
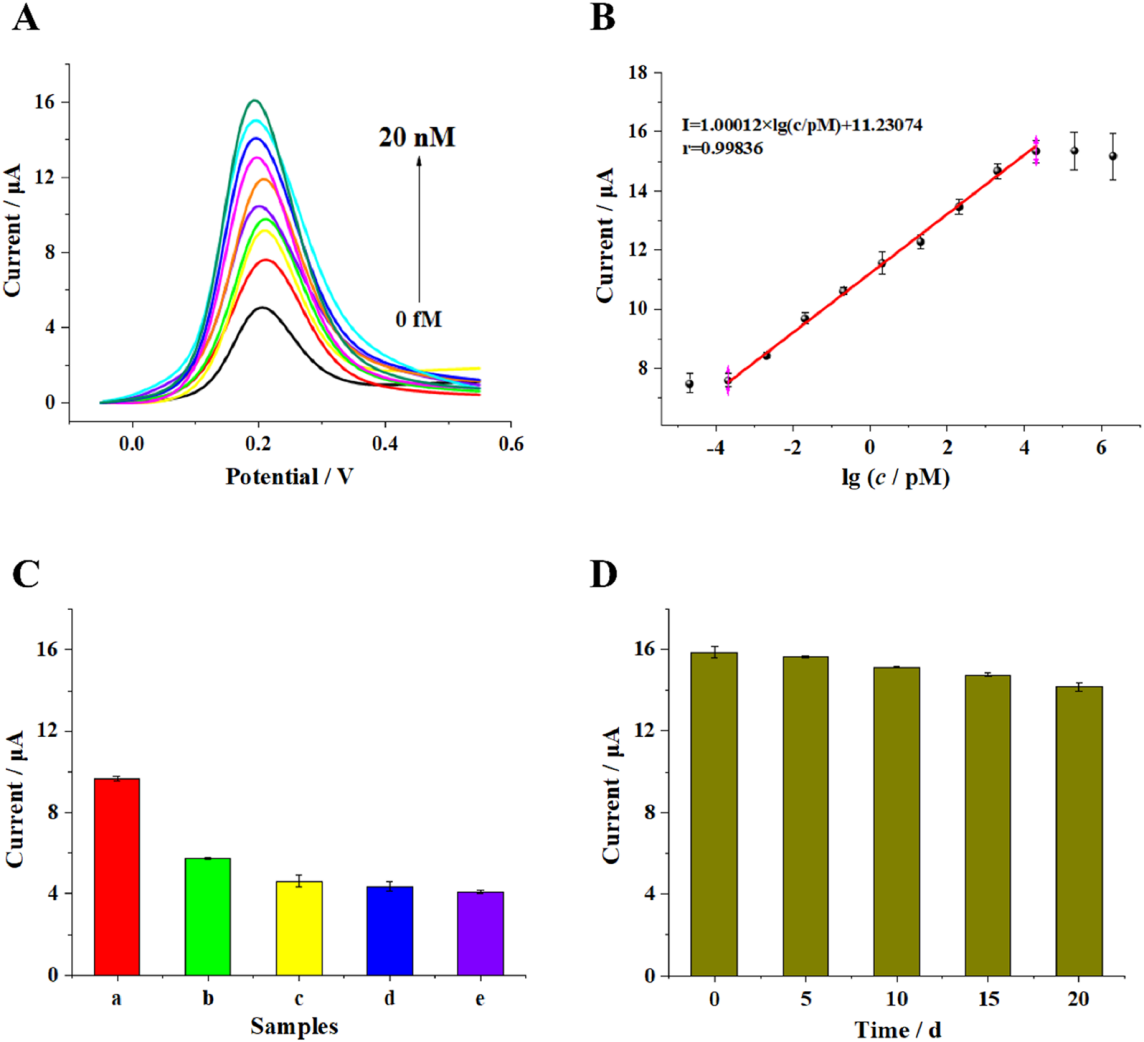
Evaluation of the sensitivity and specificity of the biosensor: **A** DPV curves response of the electrochemical biosensor upon the increase in target BCR/ABL fusion gene concentration (from bottom to top: 0 fM, 0.2 fM, 2 fM, 20 fM, 200 fM, 2 pM, 20 pM, 200 pM, and 2 nM, 20 nM, separately) and **B** the corresponding linear relationship between DPV signal and logarithmic value of target BCR/ABL fusion gene concentrations (range of concentration: 20 aM, 0.2 fM, 2 fM, 20 fM, 200 fM, 2 pM, 20 pM, 200 pM, and 2 nM, 20 nM, 200 nM, 2 uM separately). **C** DPV responses of the electrochemical biosensor to different oligonucleotides (20 fM): (a) BCR/ABL fusion gene (target), (b) single-base-mismatched strand (B1), (c) two-base-mismatched strand (B2), (d) noncomplementary strand (B3), and (e) blank. **D** Stability of the proposed biosensor. The error bars represent the standard deviation of three parallel measurements

To estimate analytical performance of the biosensor, the current responses toward BCR/ABL fusion gene at different concentrations were recorded under the optimal conditions through DPV measurements. As shown in Fig. 6A, the detection signal increased with the increasing concentration of target BCR/ABL fusion gene. The corresponding calibration plots of the peak currents showed a strong linear relationship to the logarithm value of target BCR/ABL fusion gene concentrations range from 0.2 fM to 20 nΜ with pearson correlation coefficient of 0.99836 (Fig. 6B). The linear regression equation was *I* = 1.00012 × lg (*c/*pM) + 11.23074 (*c* and *I* stood for the concentration of target BCR/ABL fusion gene and corresponding peak current value, respectively). The limit of detection was obtained based on three times the average standard deviation corresponding to blank sample detection, which was calculated to be 0.05 fM. Comparisons of this biosensor with some reported works for BCR/ABL fusion gene detection are shown in Additional file 1: Table S2, which highlighted the excellent sensitivity of this method in BCR/ABL fusion gene detection due to the cascading catalytic strategy and DNA walking machine for signal amplification.

Moreover, the specificity of the biosensor was evaluated by using 3 different DNA oligonucleotides as references, including a single-base-mismatched strand (B1), a two-base-mismatched strand (B2) and a noncomplementary strand (B3), all at concentrations of 20 fM. As depicted in Fig. 6C, the response signals of the single-base-mismatched strand and two-base-mismatched strand were much lower than the response signal of the target, revealing the good capacity of the biosensor to distinguish base-mismatch. The response signal of noncomplementary sequences were approximate to the blank solution, indicating that the biosensor presented good selectivity for DNA detection.

To evaluate the stability of the proposed biosensor, the modified electrodes were stored at 4 °C before use. As presented in Fig. 6D, there were no obvious differences during the first 5 days of storage, and the current changes were less than 1.58%. After 20 days of storage, the designed biosensor retained 89.40% of its initial current response, indicating that the proposed biosensor offers satisfactory stability for target BCR/ABL detection.

### Detection of BCR/ABL fusion gene in human serum samples

To further validate the applicability of the biosensor to complex biological matrix in clinical application, different concentrations of target BCR/ABL fusion gene were added to tenfold-diluted clinical serum samples and tested with the proposed biosensor. The detection results of BCR/ABL fusion gene in human serum samples are summarized in Additional file 1: Table S3. Satisfactory recovery values were obtained ranging from 93.60% to 110.42% with relative standard deviations (RSD) between 0.27% and 0.64%. In addition, we extracted RNA from clinical serum of BCR/ABL positive patients using spin columns CB3 according to the manufacture's protocol and tested with the proposed biosensor. The detection concentrations of BCR/ABL were compared with clinical results (by reverse transcription PCR), which were summarized in Additional file 1: Table S4. It could be seen that the proposed biosensor achieved results well matched with the clinical assay, manifesting the application potential of the proposed biosensor in clinical diagnosis.

## Conclusions

In summary, this work reported the unique catalytic activity of Ti_3_C_2_T_x_ MXene nanozyme for phenols oxidation and the application to electrochemical biosensor for BCR/ABL fusion gene detection. The catalytic activity originated from the adsorption capacity of the Ti_3_C_2_T_x_ MXene plane surface towards 1-naphthol. Theoretic calculation revealed the mechanism that relatively strong interaction was existed between the surface oxygen atoms of Ti_3_C_2_T_x_ MXene and the phenolic hydroxyl groups. Moreover, the catalytic activity was strictly proportional to the cover area of the MXene fakes but independent to the number of stacking layers, which was quite different from traditional nanozymes and significant benefited the quality control of biosensor. Ultrasensitive detection was achieved with the proposed biosensor, which provided a promising analytical tool for the clinical diagnosis of CML.

## Supplementary Information

**Additional file 1: S1**. Buffers. **S2**. Apparatus. **S3**. Synthesis of AuNPs. **S4**. Synthesis of magnetically responsive AuNPs-coated Fe3O4 (Au@Fe3O4). **S5**. Electrochemical measurement. **Fig. S1**. Energy dispersive X-ray spectroscopy (EDX) patterns of the Ti3C2Tx MXene. **Fig. S2**. DPV curves measured with bare GCE and Ti3C2Tx modified GCE with different substrates. **Fig. S3**. Optimized geometries of 1-naphthol on Ti3C2Tx MXene. **Fig. S4**. Gel electrophoresis characterization for the DNA walking strategy. **Fig. S5**. Reproducibility of the designed biosensor. **Fig. S6**. Characterization of electrode assembly process. **Table S1**. Sequences of oligonucleotides used in this work. **Table S2**. Comparison of available methods for the detection of BCR/ABL. **Table S3**. Determination of BCR/ABL in human serum samples (n=3) with the developed electrochemical biosensor. **Table S4**. Comparison of the concentration of clinical samples (n=3) by proposed method (DPV) and current widely used methods (reverse transcription PCR).

## Data Availability

All data generated or analyzed during this study are included in this article and the Additional Information.
