# Correction to: Novel Ti_3_C_2_T_x_ MXene nanozyme with manageable catalytic activity and application to electrochemical biosensor

**DOI:** 10.1186/s12951-022-01365-1

**Published:** 2022-03-26

**Authors:** Rongjun Yu, Jian Xue, Yang Wang, Jingfu Qiu, Xinyi Huang, Anyi Chen, Jianjiang Xue

**Affiliations:** 1 Department of Clinical Laboratory, University-Town Hospital of Chongqing Medical University, Chongqing, 401331 China; 2 School of Public Health and Management, Chongqing Medical University, Chongqing, 400016 China; 3 Department of Clinical Laboratory, First Affiliated Hospital of Guangxi University of Chinese Medicine, Nanning, 530023 China

## Correction to: Journal of Nanobiotechnology (2022) 20:119 https://doi.org/10.1186/s12951-022-01317-9

Following publication of the original article [1], the author reported that the corresponding authors were omitted from the author group. Dr. Xinyi Huang and Anyi Chen have been added to the author group and are presented correctly in this correction article.

There are 3 corresponding authors, including Ms. Xinyi Huang (xinyihuang1210@163.com), Dr. Anyi Chen (chenay@cqmu.edu.cn), and Prof. Dr. Jianjiang Xue (jianjiangxue@163.com).

The original article [1] has been corrected.
