# Supplementary material for: Novel Ti3C2Tx MXene nanozyme with manageable catalytic activity and application to electrochemical biosensor
Source: J Nanobiotechnology. 2022 Mar 9;20:119. doi: 10.1186/s12951-022-01317-9 (PMC8905786; doi:10.1186/s12951-022-01317-9)
Supplement: Supplementary file 1 — Additional file 1: S1. Buffers. S2. Apparatus. S3. Synthesis of AuNPs. S4. Synthesis of magnetically responsive AuNPs-coated Fe3O4 (Au@Fe3O4). S5. Electrochemical measurement. Fig. S1. Energy dispersive X-ray spectroscopy (EDX) patterns of the Ti3C2Tx MXene. Fig. S2. DPV curves measured with bare GCE and Ti3C2Tx modified GCE with different substrates. Fig. S3. Optimized geometries of 1-naphthol on Ti3C2Tx MXene. Fig. S4. Gel electrophoresis characterization for the DNA walking strategy. Fig. S5. Reproducibility of the designed biosensor. Fig. S6. Characterization of electrode assembly process. Table S1. Sequences of oligonucleotides used in this work. Table S2. Comparison of available methods for the detection of BCR/ABL. Table S3. Determination of BCR/ABL in human serum samples (n=3) with the developed electrochemical biosensor. Table S4. Comparison of the concentration of clinical samples (n=3) by proposed method (DPV) and current widely used methods (reverse transcription PCR). [file 12951_2022_1317_MOESM1_ESM.docx]

**Supporting Information for**

**Novel Ti_3_C_2_T_x_ MXene Nanozyme with Manageable Catalytic Activity and Application to Electrochemical Biosensor**

Rongjun Yu^1^, Jian Xue^2^, Yang Wang^1^, Jingfu Qiu^2^, Xinyi Huang^3,^ *, Anyi Chen ^2,^ *, Jianjiang Xue^1,^ *

*^1^ Department of Clinical Laboratory, University-Town Hospital of Chongqing Medical University, Chongqing, 401331, China*

*^2^* *School of Public Health and Management, Chongqing Medical University, Chongqing, 400016, China*

*^3^ Department of Clinical Laboratory, First Affiliated Hospital of Guangxi University of Chinese Medicine, Nanning, 530023, China*

* Corresponding authors

Tel & fax: +86-23-65714719

E-mail address: jianjiangxue@163.com (J. Xue)

**Table of contents**

**S1. Buffers**

**S2. Apparatus**

**S3. Synthesis of AuNPs**

**S4. Synthesis of magnetically responsive AuNPs-coated Fe_3_O_4_ (Au@Fe_3_O_4_)**

**S5. Electrochemical measurement**

**Fig. S1** Energy dispersive X-ray spectroscopy (EDX) patterns of the Ti_3_C_2_T_x_ MXene.

**Fig. S2** DPV curves measured with bare GCE and Ti_3_C_2_T_x_ modified GCE with different substrates.

**Fig. S3** Optimized geometries of 1-naphthol on Ti_3_C_2_T_x_ MXene.

**Fig. S4** Gel electrophoresis characterization for the DNA walking strategy.

**Fig. S5** Reproducibility of the designed biosensor.

**Fig. S6** Characterization of electrode assembly process.

**Table S1** Sequences of oligonucleotides used in this work.

**Table S2** Comparison of available methods for the detection of BCR/ABL.

**Table S3** Determination of BCR/ABL in human serum samples (n=3) with the developed electrochemical biosensor.

**Table S4** Comparison of the concentration of clinical samples (n=3) by proposed method (DPV) and current widely used methods (reverse transcription PCR).

**S1. Buffers**

The buffers and solutions involved in this experiment were as follows: DNA preparation solution (20 mM Tris-HCl containing 5 mM KCl, 140 mM NaCl, 1 mM MgCl_2_ and 1 mM CaCl_2_, pH 7.4), TCEP buffer (140 mM NaCl, 5mM KCl, 20 mM Tris-HCl and 10 mM TCEP, pH 7.4), washing buffer (Tris buffer including 20 mM Tris-HCl, 100 mM NaCl, 5 mM MgCl_2_, pH 7.4 and Tris buffer including 20 mM Tris-HCl, 100 mM NaCl, 5 mM MgCl_2_, 0.05% Tween-20, pH 7.4), DEA buffer (0.1 M diethanolamine, 100 mM KCl and 1 mM MgCl_2_, pH 9.6) and detection buffer (0.1 M DEA buffer containing 7.0 mM 1-naphthol, pH 9.6). The cyclic voltammetry (CV) and electrochemical impedance spectroscopy (EIS) were implemented in 5 mM [Fe (CN)_6_]^3-/4-^ containing 0.1 M KCl. Ultrapure distilled water (18.2 MΩ/cm) obtained from a Millipore Mill-Q purification system was used for all solution preparation.

**S2. Apparatus**

Electrochemical measurements, including differential pulse voltammetry (DPV), CV and EIS were all implemented on an AUTOLAB PGSTAT302N electrochemical workstation (Metrohm Technology Co. Ltd., Switzerland). The electrochemical workstation worked based on a conventional three-electrode system consisting of a saturated calomel electrode (reference electrode), a platinum wire (auxiliary electrode) and a 3-mm diameter glassy carbon electrode (working electrode). A JY600C electrophoresis analyzer (Beijing Junyi Electrophoresis Co. Ltd., China) was used to perform gel electrophoresis experiments, and gel images were imaged on a G: BOX F3 Gel Documentation System (Gene Co. Ltd., China). UV-vis absorption spectra of the samples were recorded on a UV-1750 UV-vis spectrophotometer (SHIMADZU Co., Ltd., Japan). Transmission electron microscopy (TEM) images were recorded by a Bruker Dimension Icon microscope (USA). Energy dispersive X-ray spectroscopy (EDX) was obtained using a JEOL JSM-6700F microscope (Japan). All electrochemical measurements were conducted at room temperature (25 °C) under ambient conditions.

**S3. Synthesis of AuNPs**

In short, sodium citrate (2 mL, 1%) was rapidly added into the boiling solution of HAuCl_4_ (100 mL, 0.01%) under magnetic stirring and maintained at 100 °C for 15 min. After the color changed from gray to wine-red, the mixture was then cooled to room temperature (RT) with continued stirring. In this way, the AuNPs had an average particle size of 15 nm.

**S4. Synthesis of magnetically responsive AuNPs-coated Fe_3_O_4_ (Au@Fe_3_O_4_)**

Magnetically responsive Au@Fe_3_O_4_ were synthesized according to the previous literature with little adjustment [1]. 500 µL NH_2_-Fe_3_O_4_ was added into the prepared 5 mL AuNPs solution and then stirred at 4 ºC for 1 h. After magnetically separating and redispersing into 1 mL PBS buffer (0.1 M, pH 7.4), the Au@Fe_3_O_4_ were obtained.

**S5. Electrochemical measurement**

The CV measurements were carried out by scanning from -0.2 V to 0.6 V at a scan rate of 100 mV s^−1^. The EIS parameters contained a 10 mV amplitude and a frequency sweep range from 10^−1^ Hz to 10^5^ Hz. The DPV measurements were executed with voltages within the range of -0.05 ~ 0.55 V, modulation amplitudes of 0.07 V, modulation time of 0.05 s and interval time of 0.2 s. All measurements were obtained under ambient conditions at room temperature (25 ± 1 °C).

**
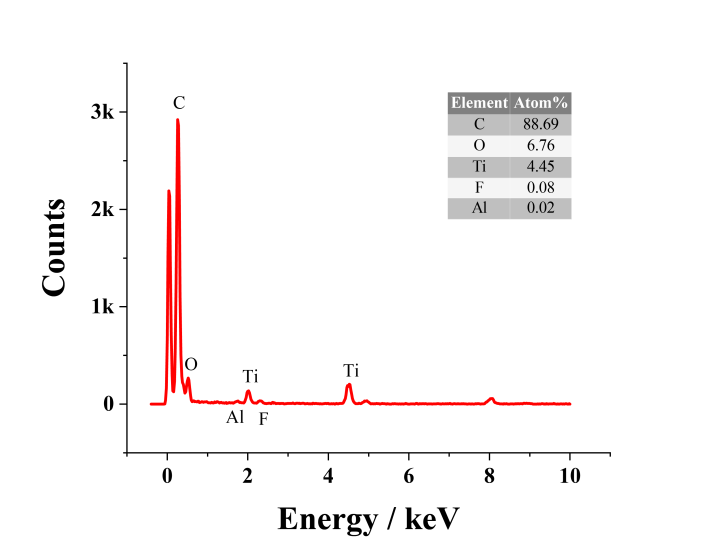
**

**Fig. S1** Energy dispersive X-ray spectroscopy (EDX) spectrum and elemental composition of the Ti_3_C_2_T_x_ MXene.


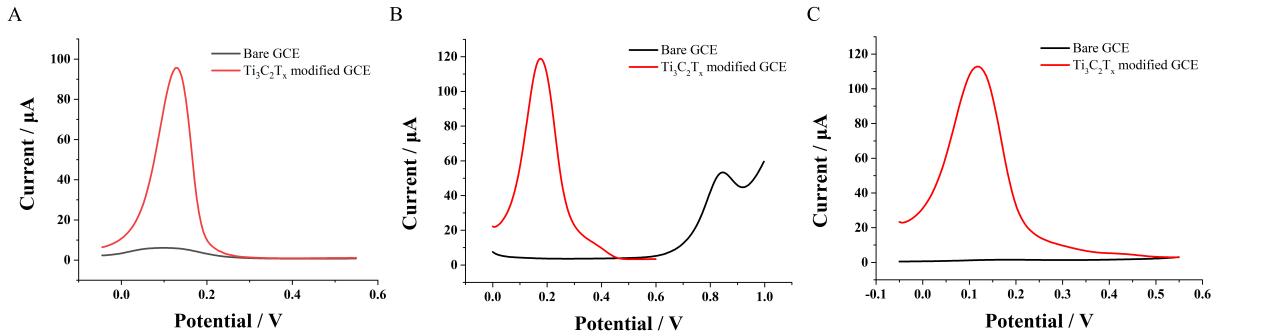


**Fig. S2** DPV curves measured with bare GCE and Ti_3_C_2_T_x_ MXene modified GCE in DEA buffers containing (A) 1-naphthol, (B) 4-nitrophenol, and (C) β-estradiol, respectively.


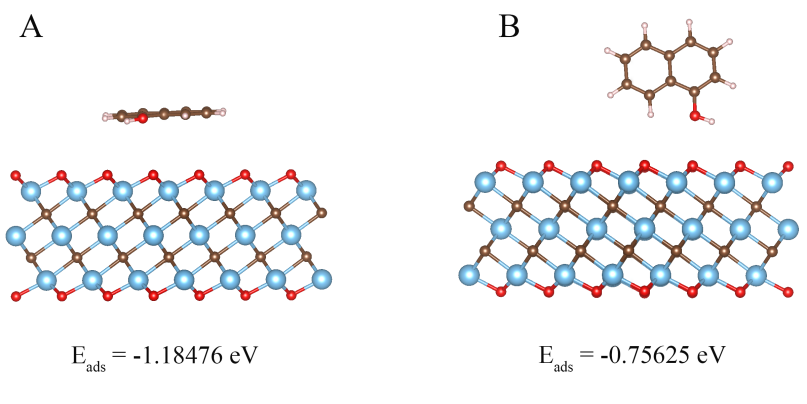


**Fig. S3** Optimized geometries of 1-naphthol on Ti_3_C_2_T_x_ MXene in (A) "lying-down" manner and (B) "standing-up" manner, respectively.

**Gel electrophoresis characterization for the DNA walking strategy**

To verify successful assembly of DNA walkers, 3.5% agarose gel electrophoresis was carried out to corroborate the interactions among these DNA sequences. As shown in Fig. S4, a single distinct band could be observed, which corresponded to the walker probe (W) (Lane 1). Then, multiple separate bands were observed from the mixture of the walker probe, support probe (S), protecting probe (P) and BCR/ABL (B) (Lane 2). As the protecting probe was first paired with the walker probe, BCR/ABL was added to pair with the protecting probe to release the walker probe, which would further pair with the support probe. Consequently, bands from top to bottom corresponded to the hybridized dsDNA of the walker probe and the support probe (walker-support dsDNA), the remaining hybridized dsDNA of the walker probe and the protecting probe, the remaining support probe and the hybridized dsDNA of the protecting probe and BCR/ABL, indicating successful hybridization among these DNA sequences. Subsequently, the walker probe was mixed with the support probe, and a clear band at a high position was observed (Lane 3), indicating the formation of walker-support dsDNA. Then, 10U Nt.BsmAI nicking endonuclease was added to the above mixture, resulting in the appearance of multiple bands (Lane 4). The band at the low position corresponded to the single stranded DNA (intermediate DNA) released from the walker-support dsDNA. In addition, agarose gel electrophoresis of dsDNA of the walker probe and protecting probe (Lane 5), dsDNA of the walker probe and protecting probe after reaction with the support probe (Lane 6), dsDNA of the protecting probe and BCR/ABL (Lane 7) is shown. Compared with lane 5, lane 6 illustrated that the support probe could not react with the walker probe in the presence of a protecting probe. The above results confirmed the interactions among these DNA sequences.


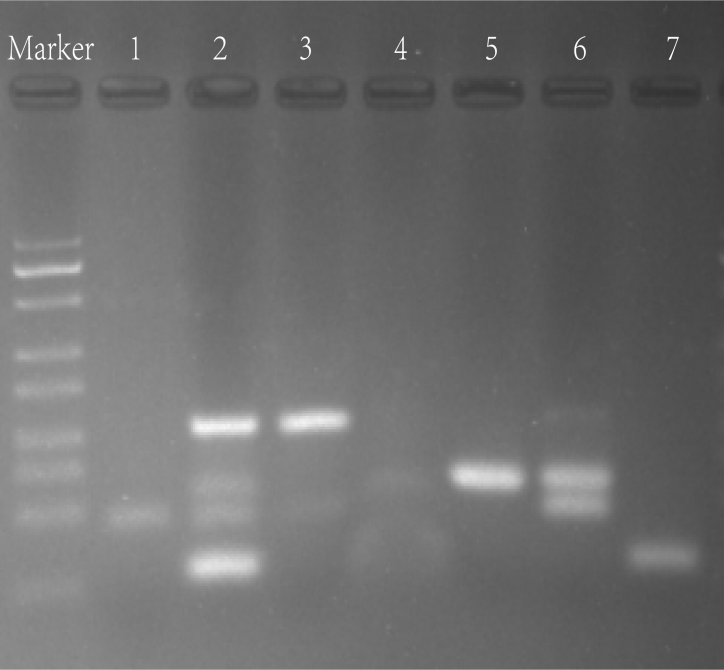


**Fig. S4** Gel electrophoresis image of different samples: (1) W, (2) W+P+S+B, (3) W+S, (4) W+P+S+B+10U Nt.BsmAI nicking endonuclease, (5) P+W, (6) P+W+S, (7) B+P. The concentrations of all DNA strands were 2.0 µM and the Maker was the sample of 20 bp DNA ladder.

**Reproducibility of the proposed biosensor**

The fabrication reproducibility of the designed biosensor was detected by measuring target BCR/ABL fusion gene at three different concentrations: 20 fM, 20 pM, and 20 nM. As illustrated in Fig. S5, after five independent measurements, these electrodes exhibited similar current responses, and the relative standard deviations (RSDs) for BCR/ABL fusion gene detection were 2.0%, 2.6%, and 1.8%, indicating that the developed biosensor had good fabrication reproducibility.


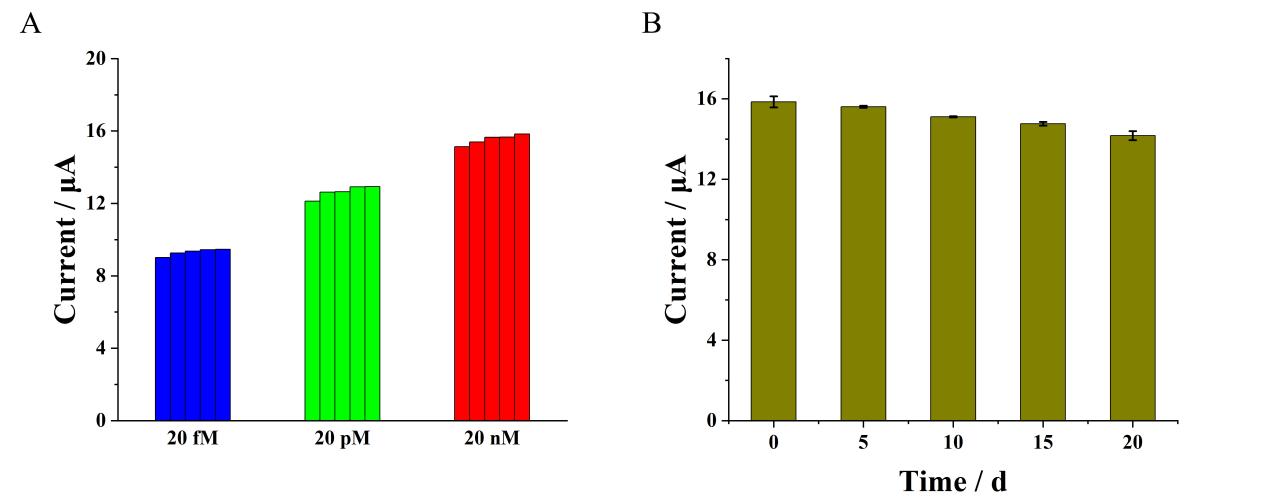


**Fig. S5.** Reproducibility of the designed biosensor: current responses of five independent measurements with 20 fM, 20 pM, and 20 nM target BCR/ABL fusion gene.

**Characterization of electrode assembly process**

To investigate the stepwise fabrication of the biosensor, cyclic voltammetry (CV) and EIS measurements were employed to characterize each stage of sensor preparation. As shown in Fig. S6A, a bare GCE curve exhibited a well-defined reversible redox peak (curve a). In curve b of the AuNPs/Ti_3_C_2_T_x_ MXene-modified electrode, the redox peak current significantly increased, indicating that the AuNPs/Ti_3_C_2_T_x_ MXene have excellent electrical conductivity and accelerate electron transfer. When the modified electrode was coated with a capture probe (CP), the peak current decreased sharply (curve c) due to the poor conductivity of the capture probe. The peak current was further decreased (curve d) when MCH was immobilized on the CP/AuNPs/ Ti_3_C_2_T_x_ MXene /GCE, which could be combined with the blocking effect of MCH to preclude electron permeation to the electrode surface. Next, the peak current continued to decrease with the modification of intermediate DNA (IP), proving the successful pairing of dsDNA (curve e). After the assembly of the biotinylated detection probe (DP), a noticeably reduced peak current could be seen; because the negatively charged DNA backbones increased from the electrode surface, the obstacle of electron transfer was increased (curve f). Finally, after the addition of SA-ALP, the peak current was reduced to a minimum value (curve g). As seen in Fig. S6B, the results obtained from EIS measurements were consistent with the outcomes of CV, where the electron transfer resistance (Ret) varied with the stepwise assembly process. These results demonstrate that each step in the fabrication of the biosensor was successful. We thank the reviewer for this kind advice.


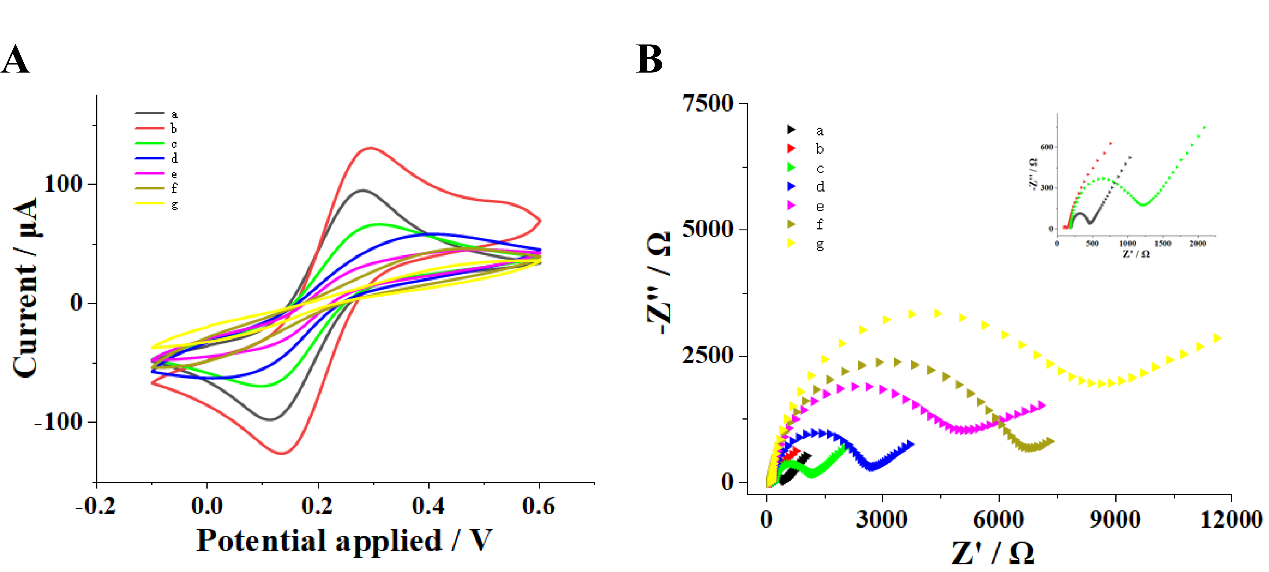


Fig. S6. Stepwise working electrode fabrication shown by the measurements of (A) CV and (B) EIS at a bare electrode (a), AuNPs/Ti_3_C_2_T_x_ MXene-modified electrode (b), CP-modified electrode (c), MCH-modified electrode (d), IP-modified electrode (e), DP-modified electrode (f) and after coupling with streptavidin-alkaline phosphatase (g). Insert: Amplified Nyquist plot of bare electrode, AuNPs/Ti_3_C_2_T_x_ MXene-modified electrode and CP-modified electrode.

**Table S1.** Sequences of oligonucleotides used in this work.

| **Oligonucleotides** | **Sequence (from 5' to 3')** |
| --- | --- |
| Walker | SH-TTTTTTGTACGCTAGACTTGACCCTCCGGCGAGACTTTGAAC TCTGCTTTTTT |
| Protecting DNA | AAAAAAGCAGAGTTCAAAAGCCCTTCAGC |
| Support DNA | SH-TTTTTTATTCATTTCAAAGTCTC*GCCGGAGGAGCGTGTGGTTGGATTGATCGTAGGTAGCTCCTCCGG |
| BCR/ABL fusion gene | GCTGAAGGGCTTTTGAACTCTG |
| Capture probe  (CP) | SH-TTTCCGGAGGAGCTACCTACGATCAATCCA |
| Detection probe | ACCACACGCTCCTCCGGCTTT-Biotin |
| Fluorescent labeled support DNA | SH-TTTTTTATTCATTTCAAAG/iBHQ1dT/CTCGCCGGAGGAGCGTGTGGTTGGATTGATCGTAGGTAGCTCCTCCGG-FAM |
| B1 | GCTGAAGGGC**A**TTTGAACTCTG |
| B2 | GCTGAAGGGC**A**TTTGA**T**CTCTG |
| B3 | ACGATAACCAGTCGTACACTAG |

B1, B2 and B3 were single-base-mismatched strand, two-base-mismatched strand and noncomplementary strand, respectively.

*: specific cleaving site of Nt.BsmAI nicking endonuclease.

**Table S2** Comparison of available methods for the detection of BCR/ABL fusion gene.

| **Detection Methods** | **Linear Range (fM)** | **LOD (fM)** | **References** |
| --- | --- | --- | --- |
| Fluorescence | 9× 10^3^ ~ 16×10^3^ | 9×10^3^ | [2] |
| Colorimetry | 1×10^3^ ~ 1×10^8^ | 1.9×10^2^ | [3] |
| Fluorescence | 1×10^6^ ~ 1.25×10^8^ | 1.5×10^5^ | [4] |
| CV and EIS | 0.0694 ~ 6.94×10^2^ | 0.0694 | [5] |
| DPV | 1× 10^4^ ~ 2×10^7^ | 1.05×10^3^ | [6] |
| **DPV** | **0.2 ~ 2×10^7^** | **0.05** | **This work** |

**Table S3** Determination of BCR/ABL in human serum samples (n=3) with the developed electrochemical biosensor.

| Sample | Added (pM) | Found (pM) | Recovery (%) | RSD (%) |
| --- | --- | --- | --- | --- |
| 1 | 0.005 | 0.00468 | 93.6 | 0.27 |
| 2 | 0.05 | 0.0479 | 95.8 | 0.35 |
| 3 | 0.5 | 0.5435 | 108.7 | 0.45 |
| 4 | 5 | 5.458 | 109.16 | 0.42 |
| 5 | 50 | 48.417 | 96.83 | 0.57 |
| 6 | 500 | 552.08 | 110.42 | 0.64 |

**Table S4** Comparison of the concentration of clinical samples (n=3) by proposed method (DPV) and current widely used methods (reverse transcription PCR).

| Sample | Concentration  (this assay, pM) | RSD (%) | Clinical result (RT-PCR, copies) |
| --- | --- | --- | --- |
| 1 | ＞2× 10^4^ | 0.57 | 735000 |
| 2 | 0.1042 | 0.87 | 1300 |
| 3 | 167.88 | 5.53 | 726000 |
| 4 | 0.03125 | 5.17 | 187 |
| 5 | 0.0168 | 2.79 | 33 |

**References**

[1] Xu Z, Liao L, Chai Y, Wang H,Yuan R. Ultrasensitive Electrochemiluminescence Biosensor for MicroRNA Detection by 3D DNA Walking Machine Based Target Conversion and Distance-Controllable Signal Quenching and Enhancing. Anal Chem 2017; 89: 8282-7.

[2] Gou X, Xu L, Yang S, Cheng X, Wu H, Zhang D, Shi W, Ding S, Zhang Y,Cheng W. One-Pot Identification of BCR/ABL Transcript Isoforms Based on Nanocluster Beacon. ACS sensors. 2021.

[3] Peng Y, Shen H, Tang S, Huang Z, Hao Y, Luo Z, Zhou F, Wang T,Feng W. Colorimetric determination of BCR/ABL fusion genes using a nanocomposite consisting of Au@Pt nanoparticles covered with a PAMAM dendrimer and acting as a peroxidase mimic. Microchim Acta. 2018; 185: 401.

[4] Shamsipur M, Nasirian V, Barati A, Mansouri K, Vaisi-Raygani A,Kashanian S. Determination of cDNA encoding BCR/ABL fusion gene in patients with chronic myelogenous leukemia using a novel FRET-based quantum dots-DNA nanosensor. Anal Chim Acta. 2017; 966: 62-70.

[5] Avelino K, Frias I A M, Lucena-Silva N, Gomes R G, de Melo C P, Oliveira M D L,Andrade C A S. Attomolar electrochemical detection of the BCR/ABL fusion gene based on an amplifying self-signal metal nanoparticle-conducting polymer hybrid composite. Colloids Surf, B. 2016; 148: 576-84.

[6] Chen X, Wang L, Sheng S, Wang T, Yang J, Xie G,Feng W. Coupling a universal DNA circuit with graphene sheets/polyaniline/AuNPs nanocomposites for the detection of BCR/ABL fusion gene. Anal Chim Acta. 2015; 889: 90-7.
